# The Effect of Chemical Surface Modification on the Repair Bond Strength of Resin Composite: An In Vitro Study

**DOI:** 10.3390/polym17040513

**Published:** 2025-02-16

**Authors:** Md Sofiqul Islam, Shadi El Bahra, Smriti Aryal A C, Vivek Padmanabhan, Abdulaziz Al Tawil, Ihab Saleh, Muhammed Mustahsen Rahman, Upoma Guha

**Affiliations:** 1 Department of Operative Dentistry, RAK College of Dental Sciences, RAK Medical and Health Sciences University, Ras Al-Khaimah P.O. Box 12973, United Arab Emirates; abdulaziz.19902008@rakmhsu.ac.ae (A.A.T.); ihab.19902042@rakmhsu.ac.ae (I.S.); 2 Department of Prosthodontics, RAK College of Dental Sciences, RAK Medical and Health Sciences University, Ras Al-Khaimah P.O. Box 12973, United Arab Emirates; shadi.elbahra@rakmhsu.ac.ae; 3 Department of Oral and Craniofacial Health Sciences, College of Dental Medicine, University of Sharjah, Sharjah P.O. Box 27272, United Arab Emirates; saryalac@sharjah.ac.ae; 4 Department of Pediatric Dentistry, RAK College of Dental Sciences, RAK Medical and Health Sciences University, Ras Al-Khaimah P.O. Box 12973, United Arab Emirates; vivek.padmanabhan@rakmhsu.ac.ae; 5 Department of Periodontology, RAK College of Dental Sciences, RAK Medical and Health Sciences University, Ras Al-Khaimah P.O. Box 12973, United Arab Emirates; mustahsen@rakmhsu.ac.ae; 6 Department of Adult Restorative Dentistry, College of Dentistry, University of Nebraska Medical Center, 4000 East Campus Loop South, Lincoln, NE 68583-0740, USA; uguha@unmc.edu

**Keywords:** clearfil tri-s bond universal quick, clearfil ap-x, silane, porcelain primer, CRB bond, shear bond strength, repair of resin composite, micro-hybrid composite

## Abstract

This in vitro study investigates the impact of the chemical modification of resin composite surfaces on repair bond strength of micro-hybrid resin composite material. First, 7 mm circular × 3 mm thick resin composite disks were prepared using teflon molds. Then, 50 specimens out of 100 were allocated for stimulated aging using a thermo-cycling (10,000 cycles) device. Both the 24 h and 1-year-aged composite discs were embedded in epoxy resin using a 2.5 cm wide × 1.5 cm thick circular mold. The surfaces were treated with Clearfil S3 bond alone or with the additional application of silane or porcelain primer. The other two groups were bonded with CRB bond with or without a porcelain primer. Using a teflon mold, a 2 mm circular and 3 mm high repair composite cylinder was built on the treated surfaces. The specimens were then stressed to de-bond by applying shear force to measure repair bond strength, and they were observed under the microscope to determine the failure pattern. The data were analyzed using SPSS26.0. Univariate analysis showed a significant effect (*p* = 0.013) of the bonding protocol on the repair bond strength; however, the effect of aging was insignificant (*p* = 0.170). The S3 bond with additional silane and the CRB bond showed the significantly higher repair bond strength of the 1-year-aged micro-hybrid composite. However, in case of 24 h aged specimens, the repair bond strength was statistically insignificant among the tested groups (*p* = 0.340). Chemical surface modification with silane has the potential to improve the repair bond strength of micro-hybrid resin composite materials.

## 1. Introduction

Resin composite materials offer several advantages over conventional dental amalgams, notably in esthetics, bonding performance and most importantly in preserving sound tooth structure. The adhesive properties of resin composite materials allow for minimal tooth preparation and, consequently, maintaining more of the healthy natural tooth structure. The micro-mechanical and chemical bonding of resin composite materials to hard tooth tissues creates a tighter seal, thereby reducing the risk of secondary caries [1]. Esthetically, resin composite materials are highly customizable, allowing for precise matching to the natural tooth shade. The color blending of resin composite with its surroundings contributes to a life-like and attractive appearance [2]. The advancements in resin composite technology have enhanced its wear resistance and durability, enabling it to withstand occlusal forces without compromising the functional and esthetic properties [3]. The chemical bonding of certain resin materials to the tooth structure further improves their stability, thus ensuring a long-lasting, reliable restoration [4]. Collectively, these factors contribute to patient satisfaction due to the esthetic outcome and functional efficiency of resin composite restoration.

Despite having good clinical performance, desirable esthetic and functional outcomes, and excellent patient satisfaction, resin composite restorations are prone to degradation over time due to various factors, including mechanical stress, hydrolytic breakdown and chemical erosion. In the oral cavity, a resin composite restoration is exposed to continuous temperature variation, pH fluctuation, and enzymatic activity, leading to the deterioration of the resin matrix and filler particles [5,6]. The water sorption by the resin matrix over time can weaken the bond between the resin and filler particle, and this causes the formation of micro-cracks and the leaching out of unreacted monomers. This phenomenon compromises the restoration’s structural integrity and esthetics [7]. Additionally, masticatory loads and mechanical debridement can wear down the restoration surface that eventually increases surface roughness and susceptibility to staining, as well as bacterial colonization. The gradual degradation of resin composite restorations reduces their longevity and effectiveness, necessitating periodic replacement or repair [8].

Repairing resin composite restorations instead of replacing them aligns seamlessly with the principle of minimally invasive (MI) dentistry. MI dentistry emphasizes the preservation of healthy natural tooth structure and conservative treatment approaches. Clinically, when a resin composite restoration displays marginal discoloration, wear, marginal degradation or a minor fracture, a repair of the defective area is highly recommended rather than the replacement of the entire restoration [9]. The clinical procedure involves the removal of the defective area and bonding fresh resin composite material to the remaining intact restoration. This MI approach minimizes the removal of the healthy tooth structure, preserves the tooth’s integrity and reduces the risk of further damage. Furthermore, this procedure, most likely, does not require anesthesia, needs less chair-side time, and helps in reducing patient’s discomfort [10]. In recent years, dental curricula have emphasized methodical education around resin restoration repair techniques to equip practitioners with the skills necessary to integrate this MI approach into routine dental care service [11].

Despite having numerous advantages and being aligned with the MI concept, achieving the optimal repair of resin composite restorations is quite challenging. There are several factors concerning the repair of resin composite restorations, including the alteration of the composite substrate surface that takes place over time due to the degradation in the oral environment. Additionally, the contamination of the substrate surface by debris and plaque reduces the bonding capability of the substrate. Secondly, the substrate surface topography plays an important role in bonding with the new repair composite material. A recently published study showed that the mechanical alteration of resin composite significantly increases the repair bond strength of resin composite [12]. Finishing and polishing resin composite restorations are essential and unavoidable steps to prevent staining, as well as to achieve the long-term color stability of the restoration [13] Therefore, it is essential to mechanically alter the substrate surface to ensure the micro-mechanical interlocking of the new repair resin composite with the old resin composite substrate.

The bonding of resin composite material to the tooth structure is based on two-fold adhesion. The first bonding interface is between the dental hard tissues and the adhesive by means of micro-mechanical interlocking (as well as chemical/ionic interaction with hydroxyapatite, if acidic functional monomers are incorporated in the chemical formula of the adhesive) [14]. The second bonding interface is between the adhesive and the resin composite by ionic exchange or co-polymerization. Unlike bonding to the tooth structure, in case of the repair procedure, the newly added composite materials should bind with the old composite substrate surface. The ability of ionic exchange and co-polymerization plays an important role for this kind of bonding. The presence of an oxygen inhibition layer (OIL) on the substrate surface is known to be a key factor for the co-polymerization of resin composite materials. However, the OIL gradually disappears from the substrate surface due to contamination and hydrolytic degradation [15].

To achieve an optimal repair bond strength, it is essential to regain the surface energy and reactivity by modifying it chemically. Nowadays, there are several types of resin composite materials routinely used by dentists. The physical, chemical and optical properties of these materials vary due to composition and variations in filler technology [16]. However, the best protocol to repair those materials has not been established yet. Thus, it is essential to evaluate the repair capability of each material following different chemical alterations of the substrate surface.

The objectives of this study were to evaluate and compare the shear bond strength (SBS) of differently aged micro-hybrid composite materials at 24 h and 1 year following different chemical surface alterations and different bonding system applications.

## 2. Materials and Methods

### 2.1. Study Design

The protocol of this in vitro study was reviewed by the research and ethical committee of Ras Al Khaimah College of Dental Sciences, and we obtained approval (RAKCODS-REC-10-2023/24-UG) prior starting the experiment.

### 2.2. Preparation of Resin Composite Substrate

Resin composite disks were prepared from a micro-hybrid composite material (Clearfil AP-X, shade A3.5 Kuraray Noritake Dental Inc, Kurasaki, Okayama, Japan). The composite material was packed into a 7 mm circular and 3 mm thick Teflon mold. To obtain a smooth surface, the packed composite was covered with a clean, scratch-free glass side, and we applied gentle pressure to maintain uniform thickness. Thereafter, a curing light was applied using a Paradigm™ Deep Cure LED Curing Light (3M Oral Care, St. Paul, MN, USA) for 40 s to polymerize the resin composite. A total of 100 specimens were prepared following this technique.

### 2.3. Sample Size Calculation

The number of required samples for this study was calculated using statistical software G*Power 3.1.9.7. With an effect size of 0.50, confidence level of 95%, and estimated power at 0.90, the number of required specimens for each groups (*n* = 10) was calculated.

### 2.4. Aging of Resin Composite Disk

To simulate the clinical scenario, the polymerized resin composite disks were divided into two groups (*n* = 50) using manual randomizer. For the short-term aging, 50 specimens were stored at 37 °C temperature in distilled water for 24 h in a temperature-controlled incubator. The long-term aging of the specimens was performed using an artificial aging device (Thermocycler THE-1100, SDMECHA-TRONIK GMBH, Feldkirchen-Westerham, Munich, Germany). To complete one cycle, the specimens were subjected to a hot thermal simulation at 55 °C and a cold thermal simulation at 50 °C in a water-submerged condition for 30 s each with a dwell time of 5 s. In total, 10,000 cycles were conducted to age the specimens by 1 year [12].

### 2.5. Embedding of Resin Composite Disks

After the completion of short-term and long-term aging processes, the specimens were embedded in an epoxy resin material. To meet the specifications of the shear bond test device, a cylindrical mold with a diameter of 25 mm and a height of 15 mm was utilized. Following the manufacturer’s instructions, the mixture of resin and its hardener was poured into the mold where the specimens were placed upside down on a level surface to prevent the flow of epoxy resin over the composite bonding surface. The embedded specimens were left in a stable position at room temperature for 48 h to ensure the complete setting of the embedding material. After retrieving the specimens from the mold, each specimen was gently wet-polished using a 2500-grit sandpaper to remove any debris attached to the specimen bonding surface.

### 2.6. Bonding Protocols

The short-term and long-term aged specimens were divided into five groups, allocating 10 specimens subjected to each type of aging (*n* = 10) using a manual randomizer. Specimen surfaces of all groups were etched with 37% phosphoric acid for 20 s and thoroughly rinsed and air-dried using a three-way syringe. After this step, specimens of group 1 (control group) were treated by applying a universal adhesive bond (CLEARFIL TRI-S BOND Universal Quick, Kuraray Noritake Dental Inc, Kurasaki, Okayama, Japan) (S3) on the bonding surfaces using a micro-brush. After a gentle air drying, the S3 adhesive bond was polymerized using a curing light as per the manufacturer’s instructions. For group 2 specimens, an additional coating with silane (Prime-Dent Silane Bond Enhancer, Prime Dental Manufacturing Inc., Chicago, IL, USA) was applied prior to the application of S3 adhesive bond. In case of group 3 specimens, a porcelain primer (Porcelain Primer, SHOFU Inc, Higashiyama-ku, Kyoto, Japan) (PP) was applied on the etched surface of the specimen as per the manufacturer’s instructions, followed by S3 adhesive bond. For groups 4 and 5, a porcelain and resin bond (CERARESIN Bond CRB, SHOFU Inc, Higashiyama-ku, Kyoto, Japan) was applied as per the manufacturer’s instructions, preceded by a pre-treatment of bonding surfaces with PP (group 4) or without PP (group 5).

### 2.7. Repair of the Substrate

Immediately after carrying out the bonding step, a 2 mm circular and 3 mm thick punched-hole Teflon mold was placed in the center of the bonded substrate and stabilized. A flowable composite material (Clearfil AP-X Flow, shade A2, Kuraray Noritake Dental Inc, Kurasaki, Okayama, Japan) was injected into the punched hole to obtain the adequate height of the repair composite cylinder. The cylinder was then polymerized for 20 s using a light cure, and the mold was gently removed. The cylinder was light cured for an additional 20 s after removing the mold.

### 2.8. Shear Bond Strength Testing

The bond strength of the repair composite compared to the short-term and long-term aged composite substrates was evaluated using shear bond strength (SBS) tests where the operator was not aware of the specific bonding protocol applied to the individual specimens. The specimens were mounted to the shear test device parallel to the direction of the semi-circular metal attachment unit that deploys the shear force. To de-bond the interface, shear stress at a crosshead speed of 1.0 mm/min was applied. The shear force in Kgf required to de-bond the test specimens were recorded. The shear bond strength value was calculated by dividing the required force by the surface area and then converting it to a megapascal (MPa) value.

### 2.9. Failure Mode Analysis

**Figure 1:**
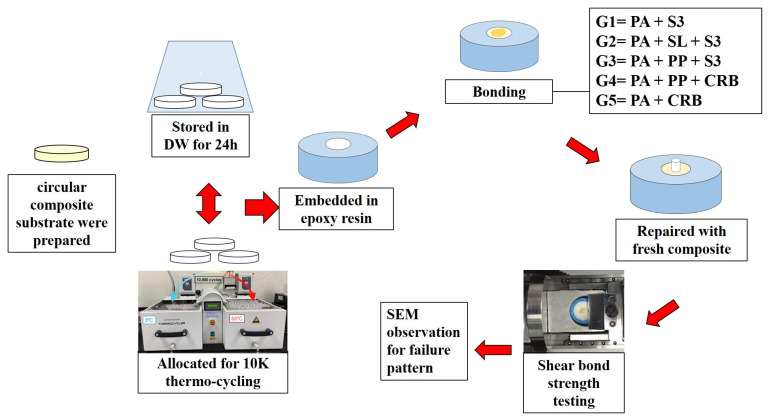
Experiment design.

After the shear bond strength test, specimens were observed at 40× magnification under a stereo microscope (Olympus BX53, Shinjuku, Tokyo, Japan) to determine the failure mode. The specimens were then dehydrated for 24 h in a desiccator and coated with 57 mm Ø × 0.1 mm thick gold/palladium (80% and 20%) in a chamber with 10^−2^ m bar vacuum pressure in the presence of argon gas and 18 mA plasma current for 120 s in a gold sputter device (Quorum Technology Mini Sputter Coater, SC7620, East Sussex, UK). After coating, specimens were observed under a scanning electron microscope (Tescan VEGA XM variable pressure SEM, Czech Republic) at the following accelerating voltage: Max. 30 kV and at 75× and 500× magnification. The study design and experimental groups are shown in Figure 1.

### 2.10. Data Analysis

The data of SBS were analyzed using statistical software (SPSS.24.0, IBM, Armonk, NY, USA). To evaluate the data distribution, descriptive statistics were established. The normality of the data was evaluated using the Shapiro–Wilk test and Kolmogorov–Smirnov test. Parametric analysis was employed based on data distribution results. Two-way ANOVA was conducted to evaluate the effect of the bonding protocol and aging on SBS. Multiple comparisons among the tested groups of short-term aged and long-term aged specimens were performed at a 95% confidence level. The bar graphs were generated using GraphPad Prism 10.2.3.

## 3. Results

### 3.1. Shear Bond Strength

**Figure 2:**
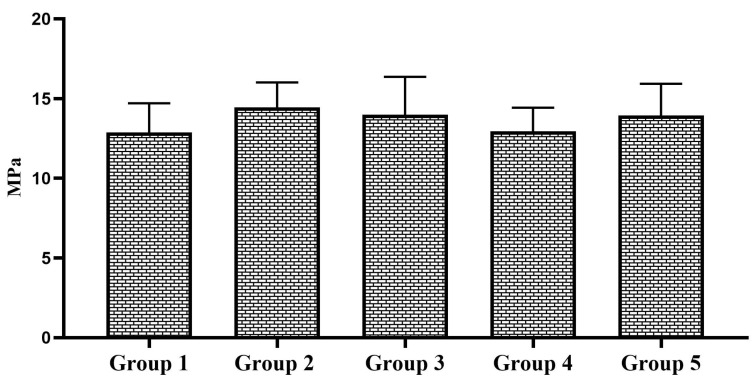
SBS of 24 h aged groups, No statistical significance observed among the tested groups.

**Figure 3:**
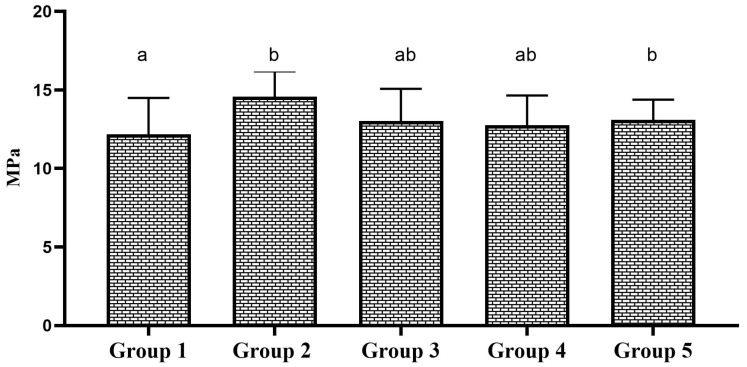
SBS of 1-year-aged groups. Bar graphs identified with same alphabet are statistically insignificant (*p* > 0.05).

**Figure 4:**
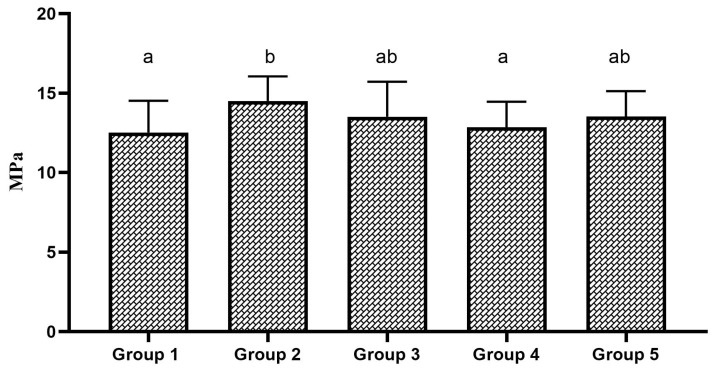
Combined SBS of 24 h and 1-year aged groups. Bar graphs identified with same alphabet are statistically insignificant (*p* > 0.05).

Two-way ANOVA showed that the type of bonding protocol has a statistically significant effect on the repair bond strength to micro-hybrid resin composite materials (*p* = 0.013); however, the aging factor did significant effect on the repair bond strength to micro-hybrid resin composite not display a statistically materials (*p* = 0.170). Multiple comparisons of 24 h aged specimens showed a statistically insignificant difference in SBS among the tested groups (*p* = 0.252). The group 2 specimens showed the highest SBS among the 24 h aged specimens. However, the SBS was statistically insignificant compared to the control group. The SBS of 24 h aged specimens is shown in Figure 2. In the case of 1-year-aged specimens, group 2 and group 5 specimens showed a statistically significant increase in repair bond strength to micro-hybrid resin composite materials compared to the control group (*p* = 0.046). The SBS of other experimental groups were statistically insignificant compared to the control group. The SBS of 1-year-aged specimens is shown in Figure 3. Considering the effect of bonding protocol regardless of aging, an additional coat of silane prior bonding (group 2) showed a statistically significant increase in repair bond strength to micro-hybrid resin composite materials compared to the control group (*p* = 0.010). The bonding protocol used for group 3 and group 5 can improve the repair bond strength; however, it does not make any significant difference compared to the control group. The effect of bonding protocol on SBS regardless of substrate aging status is shown in Figure 4.

### 3.2. Failure Pattern

**Figure 5:**
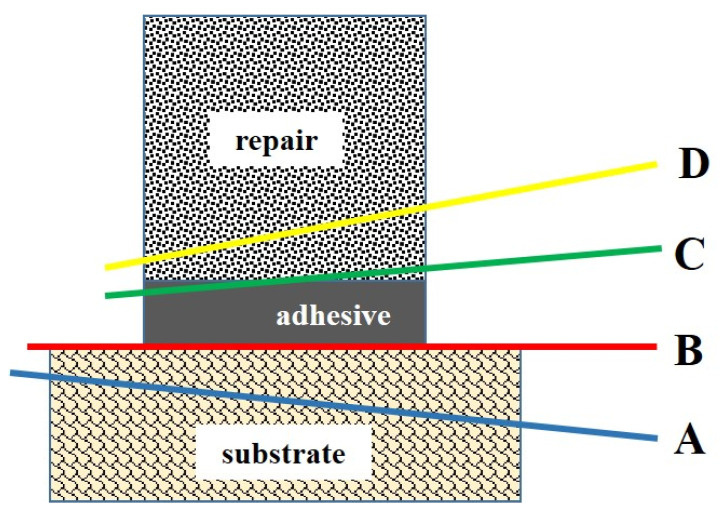
Schematic diagram of failure pattern.

**Figure 6:**
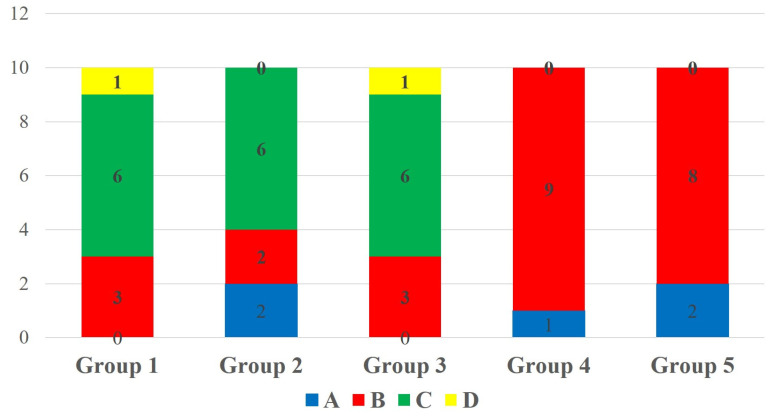
De-bond pattern of 24 h aged groups. (A) Cohesive failure within the substrate; (B) adhesive failure; (C) mixed failure; (D) cohesive failure within repair composite.

**Figure 7:**
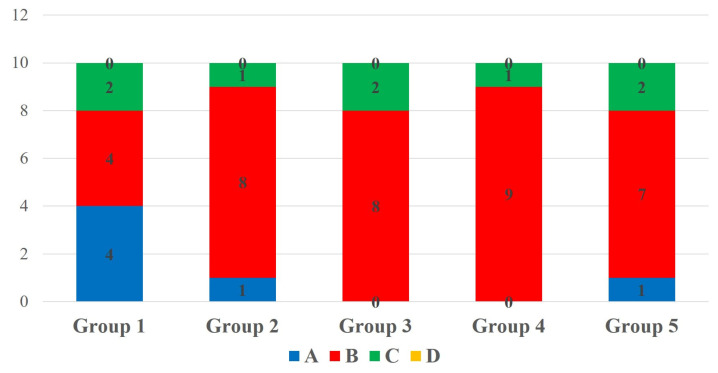
De-bond pattern of 1-year-aged groups. (A) Cohesive failure within the substrate; (B) adhesive failure; (C) mixed failure; (D) cohesive failure within repair composite.

**Figure 8:**
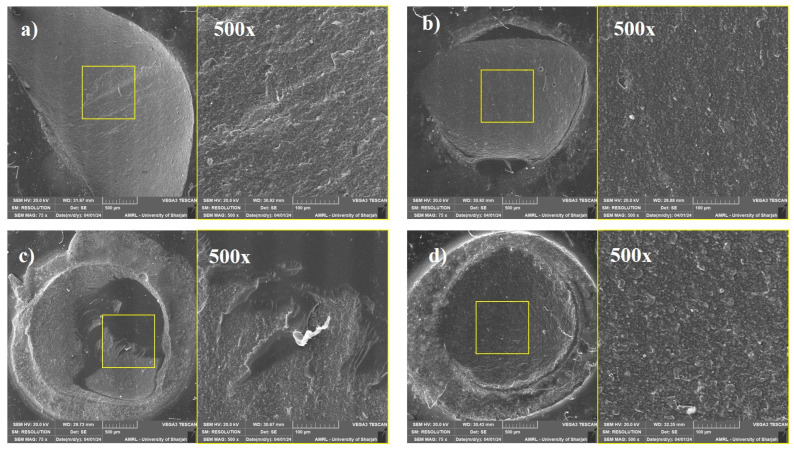
Representative SEM images of each failure pattern at 75× and 500× magnification. (**a**) cohesive failure within the substrate; (**b**) adhesive failure; (**c**) mixed failure; (**d**) cohesive failure within repair composite.

Based on microscopic observation, the failure pattern of the de-bonded specimens was classified into four categories: (A) cohesive failure within the substrate, (B) adhesive failure to the substrate, (C) mixed failure including the adhesive and the repair composite, and (D) cohesive failure within the repair composite. The fracture categories of de-bonded specimens are depicted in Figure 5. In 24 h aged specimens, C-type fracture was predominant in groups 1, 2 and 3, and B-type fracture was predominant in groups 4 and 5. The failure modes of 24 h aged specimens are depicted in Figure 6. In 1-year-aged specimens, B-type fracture was predominant in all groups except group 1 where A-type and B-type fractures were observed equally. The failure modes of 1-year-aged specimens are depicted in Figure 7. The representative SEM image of each failure pattern is shown in Figure 8.

## 4. Discussion

The current study was designed to evaluate the composite-to-composite repair bond strength using different aging conditions (24 h and 1 year) and varying bonding protocols. The thermo-cycling method was employed, which is commonly used for the artificial aging of dental materials. The shear bond strength was measured in this study, which is more relevant to clinical conditions requiring the repair of old composite materials using new ones. Moreover, we used a blinded operator to perform the SBS test, intended to eliminate the possible bias and to enhance data reliability.

The resin composite material used in this study, Clearfil AP-X, is a micro-hybrid resin composite material developed by Kuraray Noritake Dental that is renowned for its strength, radio opacity, and ease of handling [17]. The combination of aesthetic qualities with favorable mechanical properties, such as high wear and fracture resistance, rendered it a reliable choice for high-load-bearing restorations [18,19]. The resin matrix of the composite contains bisphenol-A glycidyl methacrylate (Bis-GMA) and triethylene glycol dimethacrylate (TEGDMA) monomers. The micro-sized filler particles included in the composition provide high compressive strength and minimal polymerization shrinkage, ensuring long-lasting restorations even in challenging areas. The desirable consistency of this material allows for the simple placement and precise sculpturing of the resin composite restoration.

Despite having good physical properties and bonding performance, the repair of aged resin composite restorations encounters several unique challenges. The chemical, physical, and mechanical changes that occur within the composite material over a long term of clinical service can compromise the bond strength and longevity of the subsequent repair of the composite material. Overcoming these problems requires careful surface preparation and bonding techniques selection. Composite resins become chemically inert as all free radicals used for polymerization are exhausted (depleted) over time. This phenomenon prevents the formation of a strong chemical bond between the old and new repair composite. An earlier study reported that more than 14 days are required for free radicals within a composite substrate to decline significantly [20]. The repair composite’s success relies on the available free radicals creating a covalent bond with the existing material. Continuous exposure to moisture, saliva, temperature fluctuations, and acidic environments in the oral cavity leads to the hydrolytic and thermal degradation of the resin matrix and the breakdown of the bond between the glass filler and the polymeric matrix, leading to a loss of filler particles from the surface [21]. Aged composites may lack adequate surface roughness required for micromechanical retention, especially after long-term wear and exposure. A previous study by Islam MS, et al. (2024) reported that the repair bond strength of an aged composite substrate is significantly lower than that of a non-aged one, and the mechanical roughening of an aged composite substrate can significantly improve the repair bond strength to it [12]. However, in our study the repair bond strength of 24 h and 1-year-aged substrates using CLEARFIL TRI-S BOND Universal Quick did not reveal any statistically significant difference. This result could be attributed to the structural stability of the micro-hybrid resin composite material utilized in this study or because of the superior performance of the bonding agent. CLEARFIL TRI-S BOND Universal Quick is an acrylamide-based universal bonding agent. Several studies demonstrated that acrylamide monomer-containing bonding agents exhibited an improved degree of conversion, enhanced mechanical properties, higher bond strength, and lower water sorption [22,23]. It is worth noting that Tichy, et al. (2020) found no significant impact on the micro tensile bond strength when acrylamide monomers are incorporated into the chemical formula of the universal bonding agent [24]. Nevertheless, our findings regarding the significance of aging were consistent with those reported by Tichy, et al. CLEARFIL TRI-S BOND Universal Quick contains, in addition to acrylamide monomer, 10-Methacryloyloxydecyl dihydrogen phosphate (MDP) monomer, which promotes strong chemical bonding to hydroxyapatite in enamel and dentin, resin composites, and polycrystalline zirconia ceramics. The 10-MDP monomer forms a stable, long-lasting bond to underneath substrates, and it is the most hydrolytically stable among the other acidic functional monomers included in self-adhesive adhesives/cements [25,26,27]. Furthermore, this universal adhesive bond includes silane monomer in its chemical formula. Silane is a well-known organo-functional molecule for its ability to establish a strong bond between inorganic and organic phases of the substrate and repair resin composite materials [28]. Given the synergistic effects of acrylamide, 10-MDP, silane functional monomers and the presence of other hydrophilic (HEMA) and hydrophobic (BisGMA) monomers in the chemical composition, it can be suggested that this could create a balanced, durable adhesion layer, ensuring compatibility across different bonding strategies and restorative materials.

This finding supports the versatile use of this bonding material and its potential as a repair universal bonding agent without the need for additional surface chemical treatment. Prime-Dent Silane Bond Enhancer, used in this study, is a pre-hydrolyzed silane coupling agent. A silane coupling agent is an indispensable molecule in resin composite manufacturing technology. It binds the inorganic filler particles to the organic resin matrix. Additionally, it is implemented as a priming agent to promote the chemical adhesion between the resin composite and the silica-based dental ceramic. This bifunctional compound is also capable of creating a chemical link between the incorporated silica filler particles of the existing composite and the methacrylate matrix of the new composite by means of the silanol group and methacrylate group, respectively [29]. Furthermore, silane can increase the wettability of the substrate surface by raising the surface energy. Consequently, this would facilitate the infiltration of the bonding agent into the irregularities of the substrate surface and improve the bond strength [30]. This dual action of silane enhances adhesion and ensures a strong, durable bond, especially in the repair of an existing composite where achieving an optimal result is quite challenging due to the degradation of the substrate surface. In our study, the use of silane significantly increased the SBS of the 1-year-aged substrate. This could be interpreted as the silane application satisfactorily compensating for the consequences of the aging process, which are usually manifested in a lack of free radicals and/or reactive monomers required for successful bonding to the repair resin composite [21]. Moreover, the low pH of the universal adhesive might reduce the effectiveness of the incorporated silane; therefore, the application of an additional silane layer before the adhesive bond step can be justified [31,32]. This finding is in accordance with previous studies by Çakir, N, et al., 2018 and Da Silva, C.L, et al., 2020 [33,34]. Nevertheless, the published literature reveals inconclusive results regarding the bond strength of universal adhesives with or without prior silane treatment on the bonding interface of the aged composite [35,36].

The porcelain primer used in this study is primarily designed to promote bonding between silica-containing ceramic surfaces, such as feldspathic porcelain, and resin-based materials [37]. The porcelain primer has a limited or indirect role in composite-to-composite bonding. However, it contains a silane coupling agent that might influence the bonding performance of repair resin composite materials. In our study, an additional coat with porcelain primer prior bonding showed improved SBS; however, it was statistically insignificant compared to the control group.

It is noteworthy that 24 h and 1-year-aged specimens that were pretreated with Prime-Dent Silane Bond Enhancer before applying CLEARFIL TRI-S BOND Universal Quick adhesive (group 2) exhibited higher SBS values than specimens that were pretreated with porcelain primer before applying CLEARFIL TRI-S BOND Universal Quick adhesive (group 3). This might be attributed to the chemical composition, silane molecular structure, concentration, pH, and solvent system of these two products [38].

In our study, group 5 specimens were solely treated with CERARESIN Bond CRB, which was formulated specifically for indirect restorations involving ceramic, metal, and composite surfaces. CERARESIN Bond CRB is a two-step light-cured bonding system that includes a ceramic primer containing 10-Methacryloyloxydecyl dihydrogen phosphate, which is known for its ability to create a stable chemical bond with zirconia, alumina, and other high-strength ceramic materials. In addition to 10-MDP, CERARESIN Bond CRB includes silane coupling agent, enhancing its efficacy on silica-based ceramics like lithium disilicate and feldspathic porcelain [39,40]. In case of composite repair, it showed a significantly higher shear bond strength to 1-year-aged composite substrate. The combined effect of silane and 10-MDP might be the contributor to obtain this effect. Interestingly, 24 h and 1-year-aged specimens that were pretreated with porcelain primer before applying CERARESIN Bond CRB adhesive (group 4) exhibited lower SBS values than specimens that were bonded only with CERARESIN Bond CRB adhesive (group 5).

The failure pattern of the specimens shown during bond strength testing provides important information regarding the weak point of the restoration [41,42]. In our study, the adhesive failure in 24 h aged specimens showed a negative correlation with the shear bond strength. In case of 1-year-aged specimens, the number of adhesive failures increased in all the tested groups. In the control group (without any surface modification), cohesive failure within the substrate significantly increased. This phenomenon might be associated with the degradation of the substrate surface that creates a weaker link within the restoration [43]. In our previous study, we demonstrated that mechanical surface modification has a significant role in improving the repair bond strength. The current study compared different chemical surface modification on the repair bond strength. A combination of mechanical and chemical alteration of substrate surface might help in achieving a reliable repair of composite.

These findings highlight the importance of the bonding protocol, particularly the inclusion of a silane coat, in improving the repair bond strength to micro-hybrid resin composite materials, offering valuable insights for clinical decision-making in restorative dentistry. Further investigations are warranted to optimize bonding protocols for long-term performance in various aging scenarios. One of the limitations of the current study is the in vitro study design, as the outcomes of individual clinical cases and patient-related factors might influence the results.

## 5. Conclusions

Based on the results obtained in this study, it can be concluded that the type of bonding protocol significantly influences the repair bond strength to micro-hybrid resin composite materials. The application of an additional silane coat before bonding has the potential to improve the repair bond strength of micro-hybrid composite materials regardless of aging status. Employing CLEARFIL TRI-S BOND Universal Quick with an additional silane coat before bonding or CERARESIN Bond CRB can be recommended for the repair of aged micro-hybrid resin composite.

## Data Availability

The raw data supporting the conclusions of this article will be made available by the corresponding author upon request.
